# Patterns of Melatonin Use in a Diverse National Pediatric Sample

**DOI:** 10.1001/jamanetworkopen.2024.12502

**Published:** 2024-05-22

**Authors:** Ekaterina Sadikova, Divyangana Rakesh, Henning Tiemeier

**Affiliations:** 1 Department of Social and Behavioral Sciences, Harvard T.H. Chan School of Public Health, Boston, Massachusetts; 2 Neuroimaging Department, Institute of Psychiatry, Psychology & Neuroscience, King’s College London, London, United Kingdom

## Abstract

This cohort study examines patterns of melatonin use among participants in the Adolescent Brain Cognitive Development (ABCD) Study and characterizes factors associated with use.

## Introduction

Over-the-counter melatonin sales in the US more than doubled between 2017 and 2020.^1^ While melatonin is generally considered safe, accidental ingestion reports and melatonin-related pediatric hospitalizations increased with greater sales between 2012 and 2021.^2^ Recent analysis^3^ of a demographically homogeneous convenience sample suggested that prevalence of melatonin use increases with age and may reach 19.4% by adolescence. Here, we describe longitudinal patterns of melatonin use and characterize factors associated with use in a diverse national pediatric sample.

## Methods

In this cohort study, parents of 11 864 children and adolescents aged 9 to 16 years (hereafter, children) from the Adolescent Brain Cognitive Development (ABCD) Study^4^ provided medications and supplements used by their children 2 weeks before each of 5 annual visits (2016-2022). The ABCD Study was approved by either site-specific or central (University of California, San Diego) institutional review boards. Written informed consent was obtained from the parents; assent, from the children. We followed the STROBE reporting guideline.

Child race and ethnicity were identified by parent report; categories are listed in Table 1. We compared melatonin use prevalence across ages using a linear mixed effects model and any use by race and ethnicity using a χ^2^ test. We compiled a list of child, family, and neighborhood characteristics (121 variables; missingness ranging from 0%-11% across variables was multiply imputed) to identify associations with any melatonin use and newly initiated melatonin use among treatment-naive participants at baseline. Single indicators of these outcomes were created at the end of follow-up. We used cross-validated extreme gradient boosting classification models, extracting Shapley Additive Explanation (SHAP) values to quantify relative importance of associations.^5^ Multivariable logistic regression models included the 10 factors with the highest SHAP values. Significance was tested at a 2-sided .05 level. We used R Statistical Software, version 4.1.2 (R Project for Statistical Computing). (The eMethods in Supplement 1 provides details).

**Table 1.** Prevalence of Melatonin Use by Age and by Race and Ethnicity

	Race and ethnicity, % (SD)^a^			
	Hispanic (n = 2410)	Non-Hispanic Asian (n = 252)	Non-Hispanic Black (n = 1783)	Non-Hispanic White (n = 6172)
Any use	7.9 (0.6)	3.6 (1.2)	4.9 (0.5)	15.8 (0.5)
Use by age, y				
9	1.8 (0.3)	0.0 (0.0)	1.4 (0.3)	5.2 (0.3)
10	2.5 (0.3)	0.6 (0.5)	1.6 (0.3)	5.3 (0.3)
11	2.8 (0.3)	1.9 (0.9)	1.6 (0.3)	5.4 (0.3)
12	3.2 (0.4)	2.3 (1.0)	2.1 (0.3)	6.8 (0.3)
13	4.3 (0.4)	1.2 (0.7)	1.9 (0.3)	7.1 (0.3)
14	3.0 (0.4)	1.8 (0.8)	2.8 (0.4)	8.6 (0.4)
15-16	4.5 (0.4)	0.0 (0.0)	2.3 (0.4)	6.6 (0.3)

^a^ Differences in melatonin use were statistically significant by age (*P* < .001) and by race and ethnicity (*P* < .001). Child race and ethnicity were identified by parent report. The other group (n = 1247), comprising American Indian or Alaska Native, Middle Eastern or North African, multiracial or multiethnic, and Native Hawaiian or Other Pacific Islander individuals, was omitted due to the ambiguous interpretation of prevalence in a heterogeneous group.

## Results

The study included 11 864 participants (mean [SD] age, 9.5 [0.5] years at baseline; 6163 males [51.9%] and 5701 females [48.1%]). Of these, 20.3% were Hispanic; 2.1%, non-Hispanic Asian; 15.0%, non-Hispanic Black; 52.0%, non-Hispanic White; and 10.5%, other race and ethnicity. During 5 years of follow-up, 1442 children (12.2%) used melatonin; 990 of 11 044 treatment-naive children (9.0%) initiated use after baseline. Prevalence increased significantly with age and was lowest among non-Hispanic Asian (3.6%), non-Hispanic Black (4.9%), and Hispanic (7.9%) children (*P* < .001) (Table 1). Table 2 lists the 10 factors with the highest SHAP values, given in descending order. Greater problems with initiating and maintaining sleep in children were associated with prevalent and newly initiated melatonin use (odds ratio [OR], 1.47; 95% CI, 1.39-1.56; and OR, 1.55; 95% CI, 1.47-1.64 per 1-SD change, respectively). In addition to female sex and attention problems in children, parental mental health treatment, somatic symptoms, and low parental acceptance of the child significantly predisposed to melatonin use. Melatonin use was less likely in disadvantaged neighborhoods (Table 2). Newly initiated melatonin use was associated with greater child screen time (OR, 1.04; 95% CI, 1.02-1.06 per hour), symptoms of anxiety and depression (OR, 1.09; 95% CI, 1.03-1.15 per 1-SD), and lower parental aggressive behavior scores (OR, 0.89; 95% CI, 0.84-0.95 per 1-SD).

**Table 2.** Logistic Regression Results Using the Top 10 Factors Associated With Melatonin Use, Ordered by Descending SHAP Value

Factor	Descriptive statistics		Associations^a^		
	Users	Nonusers	SHAP value	OR (95% CI)	*P* value
Problems with initiating and maintaining sleep, mean (SD), SDS subscale score^b^^,^^c^^,^^d^	13.79 (4.48)	11.47 (3.55)	0.31	1.47 (1.39-1.56)	<.001
Parent visited a doctor or counselor due to emotional or mental problem (ever vs never), No. (%)^b^^,^^c^	753 (52.2)	3111 (29.8)	0.21	1.78 (1.57-2.01)	<.001
Race and ethnicity, No. (%)^b^^,^^c^					
Hispanic	190 (13.1)	2220 (21.3)	0.18	0.59 (0.49-0.71)	<.001
Non-Hispanic Asian	9 (0.6)	243 (2.3)		0.32 (0.16-0.62)	.001
Non-Hispanic Black	88 (6.1)	1695 (16.3)		0.30 (0.23-0.39)	<.001
Non-Hispanic White	973 (67.5)	5199 (49.9)		1 [Reference]	NA
Other^e^	182 (12.6)	1065 (10.2)		0.88 (0.73-1.06)	.17
ICPSR Uniform Crime Report: adult violent crimes in the county, mean (SD), No.^b^^,^^f^	2052.35 (5237.38)	3811.28 (8007.82)	0.17	0.79 (0.72-0.87)	<.001
Child thought problems, mean (SD), CBCL raw score^c^^,^^g^	2.63 (2.85)	1.48 (2.05)	0.09	1.06 (0.99-1.14)	.08
Index of Concentration at the Extremes (income + race), mean (SD)^b^^,^^f^	0.25 (0.20)	0.19 (0.25)	0.09	1.58 (1.16-2.16)	.004
Parent somatic complaints, mean (SD), ASR t-score^b^^,^^c^	56.63 (7.13)	54.52 (5.99)	0.08	1.09 (1.03-1.15)	.002
Child attention problems, mean (SD), CBCL raw score^c^^,^^h^	4.29 (4.13)	2.80 (3.36)	0.06	1.15 (1.07-1.23)	<.001
Parental acceptance, mean (SD), Parental Behavioral Inventory Acceptance subscale score^i^	2.64 (0.40)	2.69 (0.39)	0.05	0.94 (0.89-1.00)	.048
Biological sex (female vs male), No. (%)^c^	712 (49.4)	4989 (47.9)	0.05	1.20 (1.06-1.35)	.003

Abbreviations: ASR, Adult Self-Report; CBCL, Child Behavior Checklist; ICPSR, Inter-university Consortium for Political and Social Research; NA, not applicable; OR, odds ratio; SDS, Sleep Disturbance Scale for Children; SHAP, Shapley Additive Explanation.

^a^ Area under the curve = 0.735, signaling good discrimination between those who use and do not use melatonin across ages 9 to 16 years.

^b^ Indicates variable is also in the list of 10 factors with the highest SHAP values and associated with initiating melatonin use after baseline.

^c^ Parent reported.

^d^ Scores range from 7 (no problems) to 35 (most severe problems).

^e^ Consists of individuals of American Indian or Alaska Native, Middle Eastern or North African, Native Hawaiian or Other Pacific Islander, and multiracial or multiethnic heritage.

^f^ Scores range from −1 (concentrated disadvantage) to 1 (concentrated privilege). Living in a disadvantaged neighborhood is represented by low values of Index of Concentration at the Extremes and high levels of adult violent crimes.

^g^ Scores range from 0 (none) to 18 (severe problems).

^h^ Scores range from 0 (none) to 20 (severe problems).

^i^ Youth reported. Scores range from 1 (low warmth or acceptance) to 3 (high warmth or acceptance).

## Discussion

This cohort study is the first, to our knowledge, to describe longitudinal melatonin use patterns and outline factors associated with prevalent and newly initiated melatonin use in a diverse national pediatric sample. We corroborated the age trend reported by Hartstein et al,^3^ but found lower melatonin use prevalence (12.2% in children aged 9-16 years vs 19.4% for children aged 10-13 years), likely due to differences in sample composition and melatonin use ascertainment. Despite poorer sleep,^6^ minoritized populations living in disadvantaged neighborhoods were less likely to use melatonin. While sleep health is a factor, child and parental mental health and neighborhood environments contributed significantly to melatonin uptake. A study limitation is that melatonin use was measured annually, leaving ambiguous continuity of use between visits. These findings indicate that child, family, and neighborhood determinants of use are important to consider when assessing melatonin’s safety and efficacy in the general pediatric population.

## Notes

Supplement 1.
eMethods
**eTable.** Baseline Factors Considered for Association With Any Melatonin Use and Newly Initiated Melatonin Use
eReferences

Supplement 2.
Data Sharing Statement
