# Supplementary material for: Patterns of Melatonin Use in a Diverse National Pediatric Sample
Source: JAMA Netw Open. 2024 May 22;7(5):e2412502. doi: 10.1001/jamanetworkopen.2024.12502 (PMC11112438; doi:10.1001/jamanetworkopen.2024.12502)
Supplement: Supplement 1. — eMethods eTable. Baseline Factors Considered for Association With Any Melatonin Use and Newly Initiated Melatonin Use eReferences [file jamanetwopen-e2412502-s001.pdf]

Supplementary Online Content

Sadikova E, Rakesh D, Tiemeier H. Patterns of melatonin use in a diverse national pediatric sample. *JAMA Netw Open.* 2024;7(5):e2412502. doi:10.1001/jamanetworkopen.2024.12502

eMethods

eTable. Baseline Factors Considered for Association With Any Melatonin Use and Newly Initiated Melatonin Use

eReferences

This supplementary material has been provided by the authors to give readers additional information about their work.

© 2024 Sadikova E et al. *JAMA Network Open.*

eMethods

The Adolescent Brain Cognitive Development (ABCD) Study Cohort:

ABCD was designed to characterize neuropsychiatric development from childhood to young adulthood.[1] The study aimed to recruit 11.500 children (9,780 single births and 1,720 twins) from a nationally distributed set of 21 sites across the United States, with schools sampled within the catchment area, and eligible children sampled within each school. Data collection was approved by site-specific IRBs or by a central IRB located at the University of California, San Diego.[2] Currently, the cohort at baseline includes 11,868 of the originally recruited 11,880 participants, with 12 having withdrawn consent. Although ABCD reflects diversity in U.S. socioeconomic characteristics, it is not a traditionally probability sampled study which can be used to make unbiased inferences about the U.S. population – due to the non-random allocation of the 21 recruitment sites. Recruitment was conducted from 2016 through 2018. Those with non-removable body metal (such as corrective dental braces), weight greater than the 250-pound limit for the scanners, conditions including seizure disorder, history of stroke, and cancer (among others), and a current diagnosis of schizophrenia, autism spectrum disorder (moderate or severe), intellectual disability, or alcohol/substance use disorder were excluded from study participation.

Data processing:

Baseline predictor data was assembled and multiply imputed using the fully conditional specification with predictive mean matching algorithm implemented in the *mice* R package.[3] Thirty imputed replicates were generated. At each annual visit (baseline and years 1 through 4 of follow-up), melatonin use was determined by searching all prescription and over-the-counter medication fields for case-insensitive instances of “melatonin” or “melatonex”. The available data did not outline the indication for prescription or over-the-counter purchase of any of the medications. Participants with missing melatonin information at all 5 visits (n=4) were dropped, with n=11,864 individuals included in the analysis.

Statistical models:

Differences in any melatonin use by race/ethnicity were assessed using a Chi-square test, while differences in any melatonin use across age in years was assessed by the age effect in a linear mixed effects model with random intercepts. Age at each visit, recorded in months, was rounded to the nearest year. If multiple visits occurred within the same year of age, any instance of melatonin use was attributable to that age. A set of models was run for the binary outcomes of (1) any melatonin use across the 5 visits (a single indicator per participant) and (2) the initiation of melatonin use after baseline among those who were treatment-naïve at baseline (a single indicator per participant). Within each imputation, the sample was split into 10 mutually exclusive folds of equal size, with children from the same family retained in the same fold. An Extreme Gradient Boosting model (*xgboost* R package[4]) was fit on 9 folds and a prediction was generated in the held-out fold. This cross-validation procedure repeated until each observation had an out-of-sample prediction. The following hyperparameters were specified in running *xgboost*: booster=”gbtree”, objective=”binary:logistic”, eta=0.3, gamma=0.1, max_depth=2, min_child_weight=1, subsample=1, colsample_bytree=1.Shapley Additive eXplanation (SHAP) values[5] were extracted to adjudicate relative importance of the predictors. The cross-validation procedure resulted in a SHAP value for each covariate and each individual observation based on an out-of-sample model. SHAP values can be positive or negative – indicating the direction of influence of the covariate’s value on the prediction of the outcome for the individual observation. Positive SHAP values indicate that the outcome’s prediction increases for the observation given the observed value of the covariate – and the opposite is true given negative SHAP values. Absolute values of SHAP values are averaged across all observations to estimate the marginal contribution of the covariate to the prediction of the outcome – thus quantifying the variable’s importance. For each outcome, the top 10 covaraites with the highest SHAP values were identified and used to fit a logistic regression. For further details, please refer to the following code repository: https://github.com/katsadikova/abcd_mel_use.git

Baseline factors considered for association with any melatonin use and newly initiated melatonin use:

We considered a comprehensive set of 121 characteristics describing the child (including child demographics, behaviors and activities, temperament, mental and physical health, and substance use risk), family history (including parent demographics, developmental history, and parent mental health), and culture and environment (including characterizations of the home and neighborhood environments as well as exposure to traumatic events). Please see the **eTable 1** for details.

© 2024 Sadikova E et al. *JAMA Network Open.*

eTable. Baseline Factors Considered for Association With Any Melatonin Use and Newly Initiated Melatonin Use

**Domain**	**Sub-domain**	**Variable**	**Scale/categories**	**Reporter**
Child characteristics	Demographics	Age at baseline	Years	Parent
		Sex assigned at birth	1=Male, 2=Female	Parent
		Race/ethnicity	1=non-Hispanic White, 2=non-Hispanic Black, 3=Hispanic, 4=non-Hispanic Asian, 5=Other	Parent
	Behavior and activities	Hours of screentime, typical weekday	Hours	Parent
		Hours of screentime, typical weekend day	Hours	Parent
		Participation in performing arts	1=Yes, 0=No	Parent
		Participation in visual arts	1=Yes, 0=No	Parent
		Participation in team sports	1=Yes, 0=No	Parent
		Participation in non-team sports	1=Yes, 0=No	Parent
		Participation in other activities	1=Yes, 0=No	Parent
		Participation in any organized activities	1=Yes, 0=No	Parent
	Temperament	BIS/BAS[6]: Behavioral activation - drive	Score ranging from 0 to 12	Youth
		BIS/BAS[6]: Behavioral activation - fun-seeking	Score ranging from 0 to 12	Youth
		BIS/BAS[6]: Behavioral activation - reward responsiveness	Score ranging from 0 to 15	Youth
		BIS/BAS[6]: Behavioral inhibition	Score ranging from 0 to 21	Youth
		UPPS-P[7]: Positive urgency	Score ranging from 4 to 16	Youth
		UPPS-P[7]: Negative urgency	Score ranging from 4 to 16	Youth
		UPPS-P[7]: Lack of premeditation	Score ranging from 4 to 16	Youth
		UPPS-P[7]: Lack of perseverance	Score ranging from 4 to 16	Youth
		UPPS-P[7]: Sensation-seeking	Score ranging from 4 to 16	Youth
		Prosocial behavior (parent report on youth)[8]	Score ranging from 0 to 2	Youth
		Prosocial behavior (youth report on self)[9]	Score ranging from 0 to 2	Youth
	Mental Health	Child anxious / depressed raw score (CBCL[10])	Score ranging from 0 to 26	Parent
		Child withdrawn / depressed raw score (CBCL[10])	Score ranging from 0 to 15	Parent
		Child somatic complains raw score (CBCL[10])	Score ranging from 0 to 16	Parent
		Child social problems raw score (CBCL[10])	Score ranging from 0 to 18	Parent
		Child thought problems raw score (CBCL[10])	Score ranging from 0 to 18	Parent
		Child attention problems raw score (CBCL[10])	Score ranging from 0 to 20	Parent

© 2024 Sadikova E et al. *JAMA Network Open.*

Domain	Sub-domain	Variable	Scale/categories	Reporter
		Child rule breaking raw score (CBCL[10])	Score ranging from 0 to 20	Parent
		Child aggressive behavior raw score (CBCL[10])	Score ranging from 0 to 36	Parent
		Number of friends	Count	Youth
		Number of close friends	Count	Youth
	Physical health	BMI	Derived from height and weight	Measured
		Pubertal development scale (PDS[11]) category	1=pre-pubertal, 2=early puberty, 3=mid-puberty, 4=late puberty, 5=post-pubertal	Parent
		Number of days in the past 7 physically active	Days 0-7	Youth
		Number of school days in a week with physical education	Days 0-7	Youth
		Sleep Disturbance Scale for Children[12]: Disorders of Initiating and Maintaining Sleep (DIMS)	Score ranging from 7 to 35	Parent
		Sleep Disturbance Scale for Children[12]: Sleep Breathing disorders (SBD)	Score ranging from 3 to 15	Parent
		Sleep Disturbance Scale for Children[12]: Disorder of Arousal (DA)	Score ranging from 3 to 15	Parent
		Sleep Disturbance Scale for Children[12]: Sleep-Wake transition Disorders (SWTD)	Score ranging from 6 to 30	Parent
		Sleep Disturbance Scale for Children[12]: Disorders of Excessive Somnolence (DOES)	Score ranging from 5 to 25	Parent
		Sleep Disturbance Scale for Children[12]: Sleep Hyperhydrosis (SHY)	Score ranging from 2 to 10	Parent
		Sleep Disturbance Scale for Children[12]: Total Score on sleep disturbance scale for children	Score ranging from 26 to 126	Parent
	Substance use / risk	Substance use intention: Tobacco	Score ranging from 0 to 9	Youth
		Substance use intention: Alcohol	Score ranging from 0 to 9	Youth
		Substance use intention: Marijuana	Score ranging from 0 to 9	Youth
		Substance use intention: Curious about substances	Score ranging from 0 to 9	Youth
Culture and environment	Home environment	Income-to-needs ratio (INR)	Based on US Department of Health and Human Services poverty thresholds (https://aspe.hhs.gov/topics/poverty-economic-mobility/poverty-guidelines/prior-hhs-poverty-guidelines-federal-register-references/2018-poverty-guidelines)	Parent
		Family experiences: couldn't afford food	1=Yes, 0=No	Parent
		Family experiences: couldn't afford telephone	1=Yes, 0=No	Parent
		Family experiences: couldn't afford full rent or mortgage	1=Yes, 0=No	Parent

© 2024 Sadikova E et al. *JAMA Network Open.*

Domain	Sub-domain	Variable	Scale/categories	Reporter
		Family experiences: evicted	1=Yes, 0=No	Parent
		Family experiences: utilities turned off	1=Yes, 0=No	Parent
		Family experiences: couldn't afford medical	1=Yes, 0=No	Parent
		Family experiences: couldn't afford dental	1=Yes, 0=No	Parent
		Family Environment Scale[13]: Conflict subscale score (parent-reported)	Score ranging from 0 to 9	Parent
		Family Environment Scale[13]: Conflict subscale score (youth-reported)	Score ranging from 0 to 9	Youth
		Parent behavior inventory[14] - acceptance subscale (for responding parent)	Score ranging from 1 to 3	Youth
		Parent behavior inventory[14] - acceptance subscale (for other parent)	Score ranging from 1 to 3	Youth
		Parental monitoring score[15]	Score ranging from 1 to 5	Youth
		Parent rules on substances (higher - more lax)	Score ranging from 0 to 15	Parent
	Neighborhood	SRPF[16] school environment total score	Score ranging from 6 to 24	Youth
		Continuous peer deviance score	Score ranging from 0 to 32	Youth
		At least some peer deviance	1=Yes, 0=No	Youth
		Community risk and protective factors	Score ranging from 0 to 21	Parent
		Neighborhood safety and crime (survey)[17]	Score ranging from 1 to 5	Census
		COI[18] v2.0: Nationally-normed education domain score.	Score ranging from 1 to 100	Census
		COI[18] v2.0: Nationally-normed health and environmental domain score	Score ranging from 1 to 100	Census
		COI[18] v2.0: Nationally-normed economic domain score	Score ranging from 1 to 100	Census
		Gross residential density	Density at the census tract	Census
		Census block with 2500 or more people	1 = Urbanized Area; 2 = Urban Clusters ; 3 = Rural	Census
		National Walkability Index	https://www.epa.gov/smartgrowth/national-walkability-index-user-guide-and-methodology	Census
		Traffic: Average Annual Daily Traffic	Count	Census
		Proximity to roads	Meters	Census
		Social Vulnerability Index[19]	Score ranging from 0 to 1	Census
		ICPSR Unified Crime Report: adult violent crimes in the county, per SD	Count in census tract	Census

© 2024 Sadikova E et al. *JAMA Network Open.*

Domain	Sub-domain	Variable	Scale/categories	Reporter
		Estimated lead risk in census tract	Percentage of individuals below -125 percent of poverty level in census tract of primary residential address	Census
		Total monthly average night light radiance	nW/cm2/sr	Census
		Index of Concentration at the Extremes (Income + Race)[20]	Score ranging from -1 (concentrated disadvantage) to 1 (concentrated privilege)	Census
	Traumatic events – from the K-SADS[21] post-traumatic stress disorder module	Any accident	1=Yes, 0=No	Parent
		Witnessed violence	1=Yes, 0=No	Parent
		Threat of harm/death	1=Yes, 0=No	Parent
		Physical abuse	1=Yes, 0=No	Parent
		Sexual abuse	1=Yes, 0=No	Parent
		Bullied	1=Yes, 0=No	Parent
Family history	Demographics	Parent married or living with a partner	1=Yes, 0=No	Parent
		Household income	Dollars	Parent
		Household size	Count	Parent
		Parent education (max years attained)	Years of education	Parent
	Developmental history	Birthweight	Pounds	Parent
		Maternal age at child's birth	Years	Parent
		Paternal age at child's birth	Years	Parent
		Planned pregnancy	1=Yes, 0=No	Parent
		Prenatal vitamin use	1=Yes, 0=No	Parent
		Birth complications	1=Yes, 0=No	Parent
		Premature birth	1=Yes, 0=No	Parent
		Number of days in the first 12 mo of life with fever	Count	Parent
		Number of days in the first 12 mo with serious infection / illness	Count	Parent
		Breastfeeding	Months	Parent
		Later motor development	1=Yes, 0=No	Parent
		Later speech development	1=Yes, 0=No	Parent
		Bed-wetting	1=Yes, 0=No	Parent
	Parent mental health	Father alcohol problem	1=Yes, 0=No	Parent

© 2024 Sadikova E et al. *JAMA Network Open.*

Domain	Sub-domain	Variable	Scale/categories	Reporter
		Mother alcohol problem	1=Yes, 0=No	Parent
		Mother drug problem	1=Yes, 0=No	Parent
		Father drug problem	1=Yes, 0=No	Parent
		Parent drug use for non-medicinal purposes (ASR[22])	Standardized score	Parent
		Parent: I drink too much alcohol or get drunk (ASR[22])	Standardized score	Parent
		Parent: days in last 6 months using drugs (ASR[22])	Standardized score	Parent
		Parent anxious/depressed t-score (ASR[22])	Standardized score	Parent
		Parent withdrawn t-score (ASR[22])	Standardized score	Parent
		Parent somatic complains t-score (ASR[22])	Standardized score	Parent
		Parent thought problems t-score (ASR[22])	Standardized score	Parent
		Parent attention problems t-score (ASR[22])	Standardized score	Parent
		Parent aggressive behavior t-score (ASR[22])	Standardized score	Parent
		Parent rule-breaking t-score (ASR[22])	Standardized score	Parent
		Parent intrusive t-score (ASR[22])	Standardized score	Parent
		Father seen a mental health professional	1=Yes, 0=No	Parent
		Mother seen a mental health professional	1=Yes, 0=No	Parent
		Father hospitalized for emotional/mental health problem	1=Yes, 0=No	Parent
		Mother hospitalized for emotional/mental health problem	1=Yes, 0=No	Parent

© 2024 Sadikova E et al. *JAMA Network Open.*

eReferences

1. Garavan H, Bartsch H, Conway K, et al. Recruiting the ABCD sample: Design considerations and procedures. *Dev Cogn Neurosci*. Aug 2018;32:16-22. doi:10.1016/j.dcn.2018.04.004

2. Auchter AM, Hernandez Mejia M, Heyser CJ, et al. A description of the ABCD organizational structure and communication framework. *Dev Cogn Neurosci*. Aug 2018;32:8-15. doi:10.1016/j.dcn.2018.04.003

3. van Buuren S, Groothuis-Oudshoorn K. mice: Multivariate Imputation by Chained Equations in R. *Journal of Statistical Software*. 12/12 2011;45(3):1 - 67. doi:10.18637/jss.v045.i03

4. Chen T, Guestrin C. XGBoost: A Scalable Tree Boosting System. Cornell University Library, arXiv.org; 2016:

5. Lundberg S, Lee S-I. A Unified Approach to Interpreting Model Predictions. *arXivorg*. 2017;doi:10.48550/arxiv.1705.07874

6. Carver CS, White TL. Behavioral Inhibition, Behavioral Activation, and Affective Responses to Impending Reward and Punishment: The BIS/BAS Scales. *Journal of personality and social psychology*. 1994;67(2):319-333. doi:10.1037/0022-3514.67.2.319

7. Watts AL, Smith GT, Barch DM, Sher KJ. Factor structure, measurement and structural invariance, and external validity of an abbreviated youth version of the UPPS-P Impulsive Behavior Scale. *Psychol Assess*. Apr 2020;32(4):336-347. doi:10.1037/pas0000791

8. Goodman R. The Strengths and Difficulties Questionnaire: A Research Note. *Journal of child psychology and psychiatry*. 1997;38(5):581-586. doi:10.1111/j.1469-7610.1997.tb01545.x

9. Goodman R, Meltzer H, Bailey. The strengths and difficulties questionnaire: A pilot study on the validity of the self-report version. *European child & adolescent psychiatry*. 1998;7(3):125-130. doi:10.1007/s007870050057

10. Achenbach TM. Manual for the Child Behavior Checklist/4-18 and 1991 profile. *University of Vermont, Department of Psychiatry*. 1991;

11. Kanwar P. Pubertal development and problem behaviours in Indian adolescents. *International journal of adolescence and youth*. 2020;25(1):753-764. doi:10.1080/02673843.2020.1739089

12. Bruni O, Ottaviano S, Guidetti, et al. The sleep disturbance scale for children (SDSC) construction and validation of an instrument to evaluate sleep disturbances in childhood and adolescence. *Journal of sleep research*. 1996;5(4):251-261. doi:10.1111/j.1365-2869.1996.00251.x

13. Moos RH, Moos BS. Family environment scale manual: Development, applications, research. *(No Title)*. 1994;

14. Schaefer ES. Children's reports of parental behavior: An inventory. *Child development*. 1965:413-424.

15. Karoly HC, Callahan T, Schmiege SJ, Feldstein Ewing SW. Evaluating the Hispanic paradox in the context of adolescent risky sexual behavior: The role of parent monitoring. *Journal of pediatric psychology*. 2016;41(4):429-440.

16. Arthur MW, Briney JS, Hawkins JD, Abbott RD, Brooke-Weiss BL, Catalano RF. Measuring risk and protection in communities using the Communities That Care Youth Survey. *Evaluation and program planning*. 2007;30(2):197-211.

17. Echeverria SE, Diez-Roux AV, Link BG. Reliability of self-reported neighborhood characteristics. *Journal of Urban Health*. 2004;81(4):682-701.

18. Noelke C. *The Geography of Child Opportunity: Why Neighborhoods Matter For Equity*. Institute for Child, Youth & Family Policy (ICYFP), Heller School For Social …; 2020.

19. Flanagan BE, Gregory EW, Hallisey EJ, Heitgerd JL, Lewis B. A social vulnerability index for disaster management. *Journal of homeland security and emergency management*. 2011;8(1):0000102202154773551792.

© 2024 Sadikova E et al. *JAMA Network Open.*

20. Krieger N, Kim R, Feldman J, Waterman PD. Using the Index of Concentration at the Extremes at multiple geographical levels to monitor health inequities in an era of growing spatial social polarization: Massachusetts, USA (2010–14). *International Journal of Epidemiology*. 2018;47(3):788-819. doi:10.1093/ije/dyy004

21. Geller B, Zimerman B, Williams M, et al. Reliability of the Washington University in St. Louis Kiddie Schedule for Affective Disorders and Schizophrenia (WASH-U-KSADS) mania and rapid cycling sections. *Journal of the American Academy of Child & Adolescent Psychiatry*. 2001;40(4):450-455.

22. Achenbach TM, Verhulst F. Achenbach system of empirically based assessment (ASEBA). *Burlington, Vermont*. 2010;

© 2024 Sadikova E et al. *JAMA Network Open.*
