# Supplementary material for: Patterns of Melatonin Use in a Diverse National Pediatric Sample
Source: JAMA Netw Open. 2024 May 22;7(5):e2412502. doi: 10.1001/jamanetworkopen.2024.12502 (PMC11112438; doi:10.1001/jamanetworkopen.2024.12502)
Supplement: Supplement 2. — Data Sharing Statement [file jamanetwopen-e2412502-s002.pdf]

Data Sharing Statement

Sadikova. Patterns of Melatonin Use in a Diverse National Pediatric Sample. *JAMA Netw Open*. Published May 22, 2024. doi:10.1001/jamanetworkopen.2024.12502

Data
Data available: Yes
Data types: Deidentified participant data
How to access data: Data is made available to qualified researchers who apply here: https://nda.nih.gov/abcd/request-access
When available: beginning date: 06-23-2023

Supporting Documents
Document types: Statistical/analytic code
How to access documents: The link to the repository of statistical analysis code is provided in the supplement.
When available: With publication

Additional Information
Who can access the data: Researchers whose proposed use of the data has been approved
Types of analyses: For a specified purpose
Mechanisms of data availability: With a signed data access agreement
